# Macrophage-Stimulated Cardiac Fibroblast Production of IL-6 Is Essential for TGF β/Smad Activation and Cardiac Fibrosis Induced by Angiotensin II

**DOI:** 10.1371/journal.pone.0035144

**Published:** 2012-05-04

**Authors:** Feifei Ma, Yulin Li, Lixin Jia, Yalei Han, Jizhong Cheng, Huihua Li, Yongfen Qi, Jie Du

**Affiliations:** 1 Beijing An Zhen Hospital, Affiliated to Capital Medical University, Beijing Institute of Heart, Lung, and Blood Vessel Diseases, Beijing, China; 2 The Key Laboratory of Remodeling-Related Cardiovascular Diseases, Ministry of Education, Beijing, China; 3 Department of Pathology, Capital Medical University, Beijing, China; The Chinese University of Hong Kong, Hong Kong

## Abstract

Interleukin-6 (IL-6) is an important cytokine participating in multiple biologic activities in immune regulation and inflammation. IL-6 has been associated with cardiovascular remodeling. However, the mechanism of IL-6 in hypertensive cardiac fibrosis is still unclear. Angiotensin II (Ang II) infusion in mice increased IL-6 expression in the heart. IL-6 knockout (IL-6-/-) reduced Ang II-induced cardiac fibrosis: 1) Masson trichrome staining showed that Ang II infusion significantly increased fibrotic areas of the wild-type mouse heart, which was greatly suppressed in IL-6-/- mice and 2) immunohistochemistry staining showed decreased expression of α-smooth muscle actin (α-SMA), transforming growth factor β1 (TGF-β1) and collagen I in IL-6-/- mouse heart. The baseline mRNA expression of IL-6 in cardiac fibroblasts was low and was absent in cardiomyocytes or macrophages; however, co-culture of cardiac fibroblasts with macrophages significantly increased IL-6 production and expression of α-SMA and collagen I in fibroblasts. Moreover, TGF-β1 expression and phosphorylation of TGF-β downstream signal Smad3 was stimulated by co-culture of macrophages with cardiac fibroblasts, while IL-6 neutralizing antibody decreased TGF-β1 expression and Smad3 phosphorylation in co-culture of macrophage and fibroblast. Taken together, our results indicate that macrophages stimulate cardiac fibroblasts to produce IL-6, which leads to TGF-β1 production and Smad3 phosphorylation in cardiac fibroblasts and thus stimulates cardiac fibrosis.

## Introduction

Hypertension is a multi-factorial chronic inflammatory disease. It causes cardiac remodeling, characterized by fibrosis, inflammation and hypertrophy, and it is a major cause of heart failure [1]. Activation of the rennin-angiotensin system has an important role in hypertension, cardiovascular remodeling [2], [3]. Angiotensin II (Ang II), a critical effector of this system has been implicated in the development of hypertension-induced cardiac fibrosis and inflammation [4], [5], [6].

Inflammation is a key component in the myocardial remodeling process that takes place in response to hypertension [1]. Interleukin-6 (IL-6) is a pleiotropic cytokine with a wide range of biological activities in immune regulation, hematopoiesis, and inflammation [7]. IL-6 produced by various cell types such as vascular smooth muscle cells, macrophages, fibroblasts, endothelial cells and lymphocytes, has pleiotropic effects on different organ systems. Elevated IL-6 level is associated with heart failure and is strongly prognostic of 1-year mortality [8].

A growing body of evidence suggests a close association of IL-6 level and hypertension in patients. Hirota et al. demonstrated that concomitant overexpression of IL-6 and IL-6 receptor in mice induce hypertrophy typical of that in hypertensive heart [9]. Giselleet et al. reported that IL-6 infusion cause myocardial fibrosis, hypertrophy, and diastolic dysfunction in rats [10]. However, the role of endogenous IL-6 in hypertension-induced myocardial fibrosis is unclear.

Transforming growth factor-β (TGF-β) has a pivotal role in cardiac hypertrophy and cardiac fibrosis by activating fibroblasts and producing collagen [11], [12]. TGF-β has pleiotropic effects predominantly through the intracellular mediators of TGF-β signaling, Smads2/3. Upon receptor activation, Smad2/3 are phosphorylated and form a heterotrimeric complex with Smad4 [13]. The interrelation between Ang II and TGF-β has been established. In different pathological settings and cell types, Ang II regulates TGF-β expression and activation, and the endogenous production of TGF-β mediates some Ang II responses [14]. Macrophages trigger the differentiation of fibroblasts into myofibroblasts within the infarct area after myocardial infarction, mainly through TGF-β-dependent signaling [15]. Moreover, TGF-β1-induced IL-6 expression participated in trans-differentiation of fibroblasts to myofibroblasts [16]. In the myocardium, activated myofibroblasts are the main source of extracellular matrix proteins such as collagen I/III and fibronectin [17], [18], [19].

Here we aimed to study the role of IL-6 and its possible mechanism in cardiac fibrosis induced by Ang II infusion. We found IL-6 deficiency reduced Ang II-induced cardiac fibrosis. IL-6 was mainly produced by cardiac fibroblasts by interaction with macrophages. IL-6 stimulated TGF-β1 production and Smad3 phosphorylation, which promoted differentiation of cardiac fibroblasts into myofibroblasts *in vivo* and *in vitro.*

## Materials and Methods

### Ethics Statement

Male IL-6-/- and wild-type (WT) mice in a C57BL/6 background were bred and maintained in the Laboratory of Animal Experiments at Capital Medical University. The mice were given a standard diet. The investigations conformed to the US National Institutes of Health Guide for the Care and Use of Laboratory Animals (publication no. 85-23, 1996) and were approved by the Animal Care and Use Committee of Capital Medical University.

### Animal Models

IL-6 knockout (IL-6-/-) mice on a C57/B6 background mice and wild-type (WT) littermates were purchased from the Jackson Laboratory. All mice used for experiments were within 10 to 12 weeks of age and were maintained under specific-pathogen-free conditions. The animals were handled according to the animal welfare regulations of Capital Medical University, Beijing, and all experimental procedures were approved by the Animal Subjects Committee of Capital Medical University. Male mice were randomly divided into 4 groups for treatment (n = 6 each): WT and IL-6-/-with saline infusion; WT and IL-6-/- with Ang II infusion; Hypertensive cardiac fibrosis was induced in IL-6-/- and WT mice by subcutaneous infusion of Ang II (Sigma-Aldrich, St. Louis, MO), 1500 ng/kg/min, for 7 days via an osmotic minipump (Alzet MODEL 1007D; DURECT, Cupertino, CA) as we previously described [4], [5], [6], [20], [21]. Control animals received saline infusion only. The mice were trained and Systolic blood pressure was measured by the tail-cuff system (Softron BP98A; Softron Tokyo, Japan). Data were derived from an average of 10–20 measurements per animal at each time point.

### Echocardiography

Transthoracic echocardiography was preformed on mice at day 7 after Ang II infusion. Briefly, mice were anesthetized using 3% isoflurane and then moved to a biofeedback warming station that measured heart rate, maintained core body temperature, and had nose cone anesthesia (3% isoflurane). Ultrasound gel was placed on the chest of the animal, and echocardiography measurements were obtained using a 15-mHz probe for adults and the VEVO 770 software package (Visual Sonics). M-mode tracings were taken and measured for posterior and anterior wall thicknesses, as well as the interdimensional space for both systole and diastole. Ejection fraction and fractional shortening were calculated as previously described [6], [22]. An average of three to five cardiac cycles per animal were analyzed.

### Histology and Immunohistochemistry

The ventricles were fixed in 4% paraformaldehyde and paraffin embedded hearts were sectioned at 200 µm intervals from base to apex, and serial sections of 4 µm were cut and placed on polylysine-coated glass slides. Tissue sections were deparaffinized and stained with Masson’s trichrome reagent. Immunohistochemistry involved use of a microwave-based antigen retrieval method. After the inactivation of endogenous peroxides, sections were incubated with antibodies against collagen I (Santa Cruz, 1∶600 dilution), and α-smooth muscle actin (α-SMA; both 1∶200 dilution, Abcam, Cambridge, MA), Mac-2, IL-6 or TGF-β1 (all 1∶200 dilution, Santa Cruz Biotechnology, Santa Cruz, CA) [23]. An irrelevant isotype rabbit or goat Ig G was used as a negative control (1∶200 dilution, eBioscience, CA). Digital photographs were taken at 400X magnification for more than 20 random fields from each section, and positive areas were calculated by use of NIS-ELEMENTS quantitative automatic program (NIKON, Japan) in double-blind fashion. The positive area was thresholded as a region of interest (ROI) using the hue-saturation intensity color model in the Set Color Threshold subroutine to establish the hue range of brown-stained positive to be accepted for analysis. The area of the thresholded collagen ROI was defined as the inclusive region for measurement. The area of the remaining cardiac tissue was measured as the exclusive ROI in the tissue section. The area of positive area and area of cardiac tissue in the section were recorded using the Show Regions Statistics subroutine. The percentage of positive area present in the section was determined by the ratio of inclusive area (I) of the blue-stained ROI and the exclusive area (E) of the red stained cardiac tissue ROI times 100 (% positive area [I/E] 100).

Serial transverse cryosections (7 µm thick) of frozen hearts were cut at −20°C by use of a CM1950 Frigocut (Leica, Wetzlar, Germany), placed on polylysine-coated glass slides, and stored at −80°C. Immunofluorescence with frozen hearts, 3-D frozen slices or cell-seeded samples (described below) were incubated with the primary antibodies against IL-6, DDR2 or TGF-β1 (both 1∶200 dilution, Santa Cruz Biotechnology, Santa Cruz, CA), α-SMA, F4/80 (both 1∶200 dilution, Abcam, Cambridge, MA), p-Smad3 (1∶200 dilution, Cell Signaling, Danvers, MA) and at 4°C overnight, followed by incubation with secondary antibody which are 488-donkey anti-goat and cy3-donkey anti-goat(both 1∶500 dilution, Beyotime Biotechnology, China), PE-chicken anti-rat (1∶500 dilution, Santa Cruz Biotechnology, Santa Cruz, CA), FITC-donkey anti-rabbit and 555-donkey anti-rabbit (both 1∶500 dilution, Santa Cruz Biotechnology, Santa Cruz, CA) and nuclei were counterstained with DAPI (blue). An irrelevant isotype rabbit or goat Ig G was used as a negative control (Figure S2).

### Macrophage Culture

Isolation of mouse peritoneal macrophages was as described [24]. In brief, macrophages were obtained from mice by peritoneal lavage 3 days after intraperitoneal injection with 1.5 ml sterile 3% thioglycollate. Cells were cultured in DMEM supplemented with 10% fetal bovine serum, and 100 U/ml penicillin and 100 µg/ml streptomycin. Non-adherent cells were removed after 4 h, and the medium was replaced with fresh medium every 2 days [25].

### Preparation of Primary Neonatal Cardiac Fibroblasts

Cardiac fibroblasts were isolated from neonatal IL-6-/- or WT mice (1–2 days old). Briefly, excised hearts were washed in ice-cold 1×phosphate buffer saline (1×PBS) solution, and then ventricular tissues were minced into small pieces by use of scissors in a mixture of 0.2% collagenase type 2 and 0.25% trypsin. Minced tissue was then placed into a sterile 50-ml tube and incubated with agitation at 37°C for 50 min. The supernatant was collected, and the remaining tissue was immersed in digestion solution. The supernatants were pooled and filtered through a 400-µm nylon mesh filter, then centrifuged at 1,000 g for 5 min at 4°C. The supernatant was removed, and cells were placed into fibroblast culture media (DMEM, 10% normal bovine serum and 100 U/ml penicillin/streptomycin) and incubated for 2 h at 37°C with 5% CO_2_ and 95% air. Cells were examined for positivity for vimentin. Fibroblasts between passages 6 to 12 were used for experiments.

### RNA Extraction and Real-time PCR Analysis

Total RNAs were Trizol extracted from macrophages or cardiac fibroblast isolated from IL-6-/- or WT age-matched mice according to the manufacture’s protocol (Invitrogen, Carlsbad, CA). Two micrograms of RNA was reversed transcribed by use of the MMLV and oligo dT primer method (Invitrogen). Real-time quantitative PCR reactions involved use of SYBR Green I (Takara, Otsu, Shiga, Japan). PCR amplification was performed on iQ5 Real-Time PCR Detection System (Bio-Rad, Hercules, CA) with SYBR Green JumpStart^TM^ Taq ReadyMix^TM^ (Takara, Otsu, Shiga, Japan) and specific primers for mouse IL-6, collagen I, α-SMA [4]. The housekeeping gene GAPDH or 18S RNA was used as a control. Oligo nucleotide primer sequences for each gene studied were given in Table 1.

**Table 1:** Primer sequence for Real time-PCR and RT-PCR.

mRNA	Forwards	Rewards
IL-6	5′-CTTCCATCCAGTTGCCTTCTTG-3′	5′-AATTAAGCCTCCGACTTGTGAAG-3′
α-SMA	5′-GCAAACAGGAATACGACGAAGC-3′	5′-GCTTTGGGCAGGAATGATTTG-3′
collagen I	5′-GAGCGGAGAGTACTGGATCG-3′	5′-TACTCGAACGGGAATCCATC-3′
GAPDH	5′-CCTGGAGAAACCTGCCAAGTATGA-3′	5′-AAGCAGGAATGAGAAGAGGCTGAG-3′
18S	5′-GGAAGTGCACCACCAGGAGT-3′	5′-TGCAGCCCCGGACATCTAAG-3′

### 3-D Peptide Gel Co-culture

3-D peptide gel co-culture was as described [26] with modification. Peritoneal macrophages and cardiac fibroblasts were isolated and washed with 1×PBS twice, then mixed at a ratio of 1∶1 in peptide gel at a final collagen concentration of 1.3 mg/ml. Peptide gel co-culture was maintained in DMEM containing 10% fetal bovine serum at 37°C in a humidified atmosphere containing 5% CO_2_ for 48 h. To detect the effect of IL-6 on collagen synthesis, fibroblasts and macrophages isolated from WT mice were plated on 48-well plates, serum starved for 24 h, then stimulated with IL-6 neutralizing antibody (eBioscience, San Diego, CA) at 10 µg/ml or recombinant IL-6 (rIL-6, R&D Systems) at 25 ng/ml for 48 h.

### Cytokine Analysis

To determine the production of cytokine in macrophages and fibroblasts, cells were plated at 1×10^5^ per well in 24-well plates and cultured for 48 h [27]. After incubation, the media was collected and analyzed by use of Luminex (FLEXMAP 3D) and the MILLIPLEX™ Mouse Cytokine/Chemokine kit (Millipore, Billerica, MA) to measure levels of IL-6.

### Western Blot Analysis

Fresh tissues or cells were lysed with lysis buffer (20 mM Tris(pH 7.5),1 mM EDTA,150 mM NaCl, 1 mM EGTA, 1 mM β–glycerophosphate,1% Triton X-100, 2.5 mM sodium pyrophosphate,1 mM Na_3_VO_4_, 4 µg/ml aprotinin, 4 µg/ml leupeptin, 4 µg/ml pepstatin, and 1 mM PMSF). Protein samples were separated by 10% SDS-PAGE. Nonspecific proteins were blocked by incubating the membrane with 5% nonfat dried milk in Tris-buffer saline containing 0.1% Tween 20 (TBS-T) for 1 h at room temperature with agitation. Then the nitrocellulose membrane was incubated with the primary antibodies anti-GAPDH (1∶5000 diluted in TBS-T, Kangwei, China), anti-α-SMA (1∶1000 dilution, Abcam), anti-p-Smad3 or anti-TGF-β1 (both 1∶1000 dilution, Santa Cruz Biotechnology), then with IR Dye-conjugated secondary antibodies (1∶5000, Rockland Immunochemicals, Gilbertsville, PA) for 1 h. Images were quantified by use of the Odyssey infrared imaging system (LI-COR Biosciences Lincoln, NE). The protein contents were normalized to the level of GAPDH. All experiments were repeated 3 times.

### Statistical Analysis

Data are expressed as mean ± SEM. Differences between groups were analyzed by Student’s *t* test or ANOVA, then Newman-Keuls Multiple Comparison Test by using Graphpad Software (GraphPad Prism version 5.0 for windows, Graphpad Software). *P<*0.05 was considered statistically significant.

## Results

### Increased IL-6 Expression in Ang II-induced Cardiac Fibrosis

**Figure 1:**
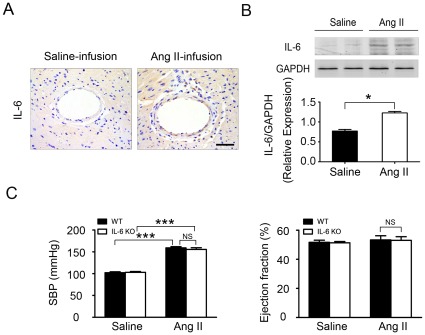
IL-6 production is increased in cardiac fibrosis induced by Ang II. (**A**) Immunohistochemical analysis of IL-6 production (brown) in ventricles of mice (n = 6 per group) after saline or Ang II infusion. (**B**) Western blot analysis for IL-6 expression in the hearts of WT mice. (**C**) Systolic blood pressure (SBP) was measured and echocardiography was performed at day 7 after Ang II infusion. Error bars represent mean±SEM. *P<0.05; ***P<0.001. Scale bars: 50 µm.

To establish the role of IL-6 in the development of Ang II–induced cardiac remodeling, WT mice were infused with Ang II, at 1500 ng/kg/min for 7 days [5], [6], [20], [21], and IL-6 expression in myocardium was determined by immunohistochemistry and western blot analysis. Ang II infusion significantly increased IL-6 expression in the myocardium as compared with saline infusion controls (Figure 1A and B). As well, Ang II infusion elevated systolic blood pressure in both WT and IL-6-/- groups as compared with saline-treated mice, with no difference between WT and IL-6-/- mice (Figure 1C) [20]. The 2 experimental groups did not differ in cardiac function characterized by left ventricular ejection fraction (Figure 1C) and fractional shortening or ratio of heart to body weight or after Ang II infusion (Figure S1), which indicated that IL-6 expression deficiency did not affect blood pressure or heart function after a short time period Ang II infusion (at day 7).

### IL-6 Deficiency Decreases Cardiac Fibrosis

**Figure 2:**
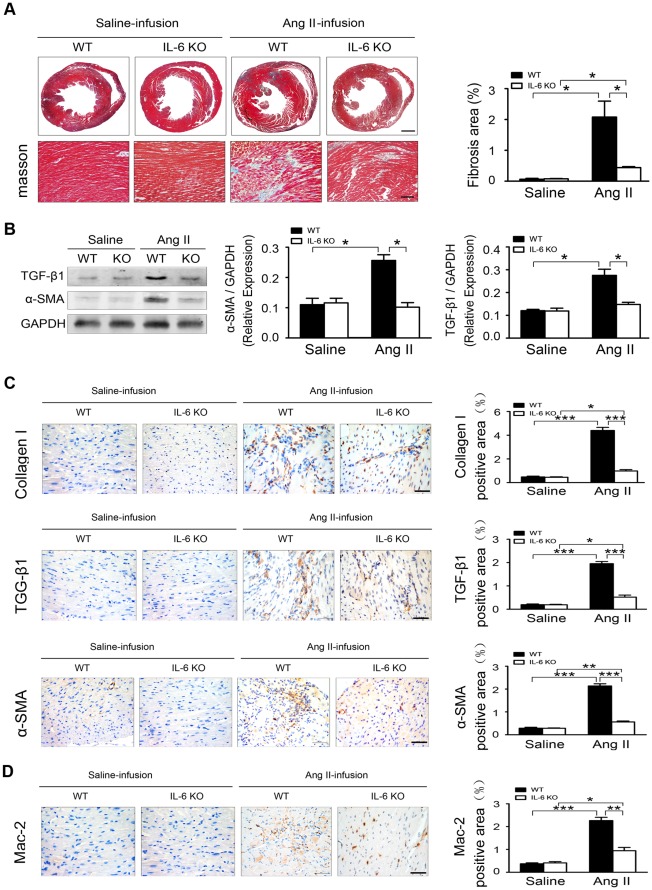
IL-6 promotes Ang II-induced cardiac fibrosis and inflammation. (**A**) Ang II-induced hypertensive cardiac fibrosis in C57BL/6 wild-type (WT) and IL-6-/- mice (n = 6 per group). (Left) Masson’s trichrome staining (blue) of ventricular sections analyzed for fibrotic areas at day 7 after Ang II infusion (n = 6 mice/group). Scale bars: 500 µm (top), 100 µm (bottom). Fibrotic area is stained blue. (Right) Quantification of fibrotic areas. (**B**) Western blot analysis and quantification of protein levels of transforming growth factor β1 (TGF-β1) and α-smooth muscle actin (α-SMA) in WT and IL-6-/- hearts (n = 6 per group). Normalization is to GAPDH level. (**C**) Representative immunohistochemical staining of collagen I, TGF-β1 and α-SMA in WT and IL-6-/- hearts. Scale bars: 50 µm and quantification (right). (**D**) Immunohistochemical staining and quantification of Mac-2 in WT and IL-6-/- hearts. Scale bars: 50 µm. Data represent the mean±SEM (n = 5 per group). *P<0.05; **P<0.01; ***P<0.001.

Because inflammation plays an important role in myocardial remodeling in response to hypertension and exogenous IL-6 may cause myocardial fibrosis [10], we examined the effect of endogenous IL-6 on Ang II-induced cardiac fibrosis. Masson trichrome staining showed fibrotic areas were significantly greater in WT than IL-6-/- hearts with Ang II infusion (Figure 2A). Western blot detected protein levels of transforming growth factor β1 (TGF-β1) and α-smooth muscle actin (α-SMA) were significantly greater in WT than IL-6-/- hearts with Ang II infusion (Figure 2B). Immunohistochemistry confirmed the expression of collagen I, a major component of fibrotic lesions; TGF-β1, a pro-fibrotic cytokine; and α-SMA, a marker of differentiation of fibroblasts into myofibroblasts, were all markedly lower in IL-6-/- mice than that of in WT mice after Ang II infusion (Figure 2C). Therefore, endogenous IL-6 plays a critical role in cardiac fibrosis in response to Ang II infusion. The number of the macrophage cell marker Mac-2 positive cells was lower in IL-6-/- than WT mice with Ang II infusion (Figure 2D), suggesting IL-6 deficiency decreased inflammation and cardiac fibrosis induced by Ang II.

### IL-6 is Mainly Produced by Cardiac Fibroblasts in Response to Ang II Stimulation

**Figure 3:**
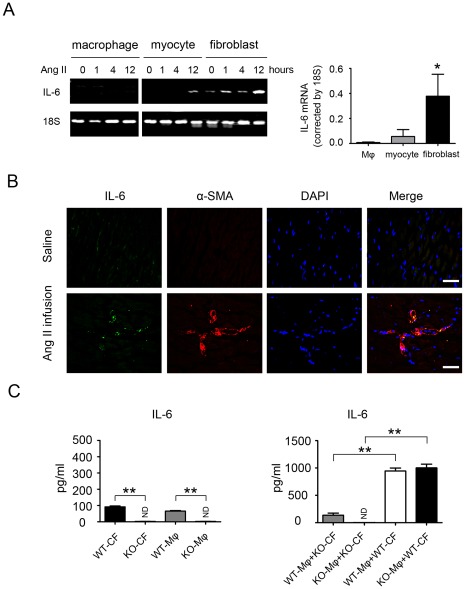
Generation of inflammatory factors and localization of IL-6 in macrophages or myofibroblasts in Ang II-infused mice. (**A**) RT-PCR analysis of mRNA expression of IL-6 in WT mice macrophages, cardiomyocytes and cardiac fibroblasts with Ang II treatment at 0,1,4,12 h respectively. *P<0.05 vs. macrophages. (**B**) IL-6 (green) was detected in WT hearts and in myofibroblasts (red) in Ang II-treated hearts. Nuclei are DAPI stained (blue; right). (**C**) Macrophages (Mφ) were co-cultured with cardiac fibroblasts (CF), and the level of IL-6 production in the medium was measured by luminex assay (n = 5 per group). *P<0.05; ND: no detective; **P<0.01 vs. WT macrophages (Mφ(WT)) and cardiac fibroblasts(CF(WT)); **P<0.01 vs. Mφ(WT)+cardiac fibroblasts CF(IL-6-/-) and Mφ(IL-6-/-)+CF(IL-6-/-).

We next evaluated the cellular source of IL-6 in Ang II-induced cardiac fibrosis. Cardiomyocytes, macrophages, or fibroblasts derived from WT mouse heart were treated with Ang II (100 nmol/L) for various time (0,1,4,12 h respectively). RT-PCR revealed the IL-6 expression was primarily present in cardiac fibroblasts (Figure 3A). Furthermore, immunofluorescence revealed significantly greater IL-6 level in α-SMA–positive myofibroblasts (Figure 3B) than that in F4/80-positive macrophages with Ang II treatment (Figure S3). Luminex analysis showed that either cardiac fibroblasts or macrophages alone produce low level IL-6 (less than 100 pg/ml of supernatant at 48 h, Figure 3C, left panel), however when cardiac fibroblasts and macrophages were co-cultured together, IL-6 production were significantly increased by 10.5 folds compared to macrophages or cardiac fibroblasts alone (Figure 3C, right panel). Importantly, co-culture of WT macrophages with IL-6-/- cardiac fibroblasts produced very low amounts of IL-6, but co-culture of IL-6-/- macrophages with WT cardiac fibroblasts was still able to produce high level IL-6 (Figure 3C, right panel). Therefore, endogenous IL-6 is mainly produced by cardiac fibroblasts in a macrophage-dependent manner in response to Ang II infusion.

### IL-6 Promotes Cardiac Fibroblast Collagen Synthesis and Fibroblast Activation *in vitro*

**Figure 4:**
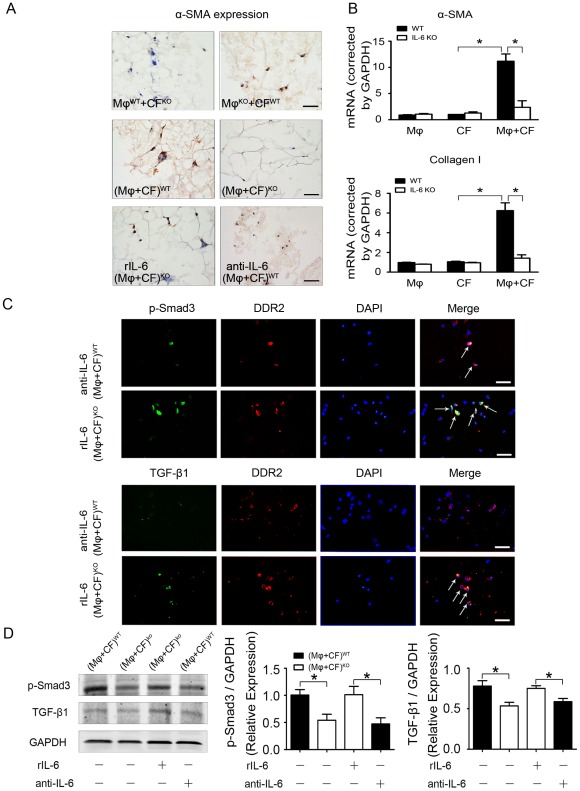
Co-culture of macrophages and myofibroblasts induces α-SMA, TGF-β1 and Smad3 expression. (**A**) Representative immunohistochemical staining of α-SMA with 3-D co-culture in the present of Ang II. Scale bars: 50 µm. (**B**) Real-time PCR quantification of α-SMA and TGF-β1 mRNA expression. (**C**) Immunofluorescence staining of protein level of phosphorylated Smad3 (p-Smad3; green) and DDR2 (red) or Immunofluorescence staining of TGF-β1 (green) and DDR2 (red) in co-cultured WT macrophages and fibroblasts with neutralizing antibody to IL-6 and co-cultured IL-6-/- macrophages and fibroblasts with recombinant IL-6 (rIL-6) treatment. Scale bars: 50 µm. (n = 6 per group). (**D**) Western blot analysis and quantification of protein level of TGF-β1 and p-Smad3 in 3-D co-culture (n = 4 per group). *P<0.05.

To delineate how endogenous IL-6 regulates cardiac fibrosis, we next determined its role in fibroblast activation in a co-culture system of cardiac fibroblasts and macrophages. The expression of α-SMA (a marker of myofibroblasts) was examined in 3-D co-culture of cardiac fibroblasts with macrophages stimulated by Ang II. Cardiac fibroblasts or macrophages alone cultured in 3-D peptide gel did not express α-SMA (Figure S4A), however, co-culture of WT cardiac fibroblasts with IL-6 KO macrophages stimulated α-SMA expression, whereas co-culture of IL-6 KO cardiac fibroblasts with WT macrophages expressed very low-level α-SMA expression (Figure 4A, top panel). Co-culture of WT cardiac fibroblasts with macrophages stimulated α-SMA expression, whereas co-culture of IL-6 KO cardiac fibroblasts with macrophages did not express α-SMA expression (Figure 4A, middle panel). Treatment of co-culture of IL-6 KO cardiac fibroblasts and macrophages with recombinant IL-6 restored the expression of α-SMA while treatment of co-culture of WT cells with neutralizing IL-6 antibody reduced the expression of α-SMA (Figure 4A, lower panel). Real-time PCR revealed that co-culture of cardiac fibroblasts with macrophages promoted the production of α-SMA and collagen, indicating cardiac fibroblasts activation was depended on macrophages (Figure 4B). Immunofluorescence staining showed that IL-6 induced the phosphorylation of Smad3 and TGF-β1 expression. Immunofluorescence staining showed that cultured WT fibroblasts and IL-6-/- fibroblasts alone almost did not express phosphorylation of Smad3 and TGF-β1 (Figure S4B). Administration of neutralizing antibody to IL-6 to the co-culture of fibroblasts and macrophages, phosphorylation of Smad3 and TGF-β1 expression were reduced (Figure 4C, top panel). Treatment of co-culture of IL-6 KO macrophages and cardiac fibroblasts in 3-D peptide gels with IL-6 also increased phosphorylation of Smad3 and TGF-β1 expression (Figure 4C, lower panel). Finally, western blot analysis verified IL-6 stimulated the phosphorylation of Smad3 and TGF-β1 expression (Figure 4D).

## Discussion

Both experimental studies and epidemiology showed that the inflammatory response plays a critical role in hypertension-induced cardiac remodeling. However, the relative importance of specific cytokines in the pathogenesis of hypertension has yet to be fully elucidated. We studied the role of IL-6 in cardiac fibrosis induced by Ang II infusion in mice. Ang II infusion increased IL-6 expression in hearts of mice, and IL-6 was mainly produced by cardiac fibroblasts through interaction with macrophages. Macrophage-stimulated cardiac-fibroblast production of IL-6 induced TGF-β1 production and Smad3 phosphorylation in cardiac fibroblasts, which promoted the differentiation of cardiac fibroblasts into myofibroblasts *in vivo* and *in vitro.* Finally, IL-6 deficiency reduced Ang II-induced cardiac inflammation and fibrosis.

Clinical trials demonstrated a correlation of increased levels of circulating IL-6 in patients and heart failure severity and mortality [8]. IL-6 directly mediated myocardial fibrosis, hypertrophy, and diastolic dysfunction [10]. However, the role of endogenous IL-6 in hypertension-induced cardiac fibrosis was unclear, and the cell type producing IL-6 was unknown. We showed that Ang II infusion significantly increased IL-6 expression in the myocardium (Figure 1). It has been shown that IL-6 directly mediated myocardial fibrosis by inducing a conversion in fibroblast phenotype to myofibroblasts [10]. Cardiac fibroblasts stimulated with Ang II were found to secrete members of the IL-6 family, including IL-6 itself, which induced cardiomyocyte hypertrophy [28]. We found that in the WT heart, Ang II infusion significantly increased the fibrotic area, which was greatly suppressed in IL-6-/- mice (Figure 2). Immunohistochemistry revealed the expression of collagen I, a major component of fibrotic lesions; TGF-β1, a pro-fibrotic cytokine; and α-SMA, a marker of differentiation of fibroblasts into myofibroblasts, all markedly decreased in IL-6-/- mice (Figure 2C), which suggests that endogenous IL-6 plays a critical role in cardiac fibrosis in response to Ang II infusion. Concurrently, the level of the inflammatory cell marker Mac-2 was greatly reduced in IL-6-/- mice (Figure 2D), indicating that IL-6 deficiency decreases inflammation and cardiac fibrosis induced by Ang II. Even though Ang II infusion for 7 days induced IL-6, TGF-β1, collagen I and fibrosis in the heart (Figure 2), but did no change blood pressure or cardiac function (Figure 1C). Our results are consistent with other studies that have shown that knockout of IL-6 do not change Ang II-induced blood pressure increases [20], [29]. Several studies have shown that long term Ang II infusion (over 4 weeks,) caused cardiac hypertrophy, remodeling and cardiac dysfunction in mice [30], [31], however, short term infusion of Ang II infusion (7 days) was not enough to cause cardiomyocyte hypertrophy thus cardiac dysfunction, but instead, caused cardiac inflammation. IL-6 is produced by various cell types such as vascular smooth muscle cells, macrophages, fibroblasts, endothelial cells or lymphocytes, and has pleiotropic effects on different organ systems [32]. The diversity of IL-6–dependent effects is widespread. Elevated IL-6 serum levels have been detected in acute and chronic inflammation, and IL-6 directly induces cardiac fibrosis, concentric hypertrophy, and diastolic dysfunction in rats [10]. However, the cell type producing endogenous IL-6 in cardiac remodeling was unclear. Immunofluorescence staining revealed significantly greater IL-6 accumulation in α-SMA–positive myofibroblasts (Figure 3B) than in F4/80–positive macrophages in Ang II-induced cardiac fibrosis *in vivo* (Figure S3). Moreover, *in vitro* evidence showed that isolated cardiac fibroblasts expressed a low level of IL-6 while macrophages or cardiomyocytes barely have IL-6. IL-6 expression was significantly increased in cardiac fibroblasts when WT or IL-6-/- macrophages were present, however, there was very low IL-6 expression in IL-6-/- cardiac fibroblasts even WT or IL-6-/- macrophages were present (Figure 3C). Therefore, endogenous IL-6 is mainly produced by cardiac fibroblasts in Ang II-induced cardiac fibrosis and it is macrophage dependent.

Cardiac fibrosis is mediated in part by TGF-β1, a potent stimulator of collagen-producing cardiac fibroblasts [11], [33], [34]. In cultured fibroblasts, TGF-β activates fibroblasts and induces the production of growth factors, angiogenic factors, extracellular matrix proteins, and proteases [35]. Macrophages trigger the differentiation of fibroblasts into myofibroblasts within the infarct area, mainly through TGF-β–dependent signaling in the ischemic myocardium [15]. Our results showed the level of the pro-fibrotic cytokine TGF-β1 markedly decreased in IL-6-/- mice treated with Ang II, and rIL-6 increased TGF-β1 production (Figure 4). TGF-β1 has pleiotropic effects predominantly through intracellular mediators of TGF-β signaling such as Smads2/3 [36]. Our results showed that IL-6 induced the phosphorylation of Smad3 and TGF-β1 expression, which was reduced with neutralizing IL-6 antibody. 3D co-culture demonstrated that increased phosphorylation of Smad3 and α-SMA expression was depended on IL-6, which suggests that macrophages might have a synergetic role with IL-6 in triggering the differentiation of cardiac fibroblasts into myofibroblasts, mediated in part by a TGF-β1/Smad3 pathway (Figure 4). We showed in the present study that Ang II induces IL-6 and fibrosis, which might be mediated in part by the TGF-β1/Smad3 pathway, this conclusion is consistent with other studies. Aoki et al. reported that there was an autocrine loop between IL-6 and TGF-β1 through Smad2/3-dependent pathways in activated pancreatic stellate cells. Pancreatic stellate cells express and secrete IL-6, while anti-TGF-β1 neutralizing antibody blocked IL-6 secretion. Moreover, anti-IL-6 neutralizing antibody inhibited TGF-β1 secretion from PSCs [37]. In another study, Zhang et al. showed that TGF-β1-stimulated Smad-responsive promoter transcriptional activity was significantly greater in the presence of IL-6 than that induced by TGF-β1 alone. Augmented TGF-β1/Smad signaling following the addition of IL-6 appeared to be mediated through binding to the cognate IL-6 receptor [38].

In summary, IL-6 expression was increased in Ang II-infused mouse heart, and IL-6 deficiency ameliorated cardiac inflammation and fibrosis. IL-6 is mainly produced by cardiac fibroblasts with Ang II stimulation and is macrophage dependent; the production induces collagen synthesis in cardiac fibroblasts and promotes differentiation of fibroblasts into myofibroblasts *in vivo* and *in vitro*. The pro-fibrotic effects of IL-6 might be mediated in part by the TGF-β1/Smad3 pathway.

## Supporting Information

Figure S1 **Quantification of body weight and heart/body weight from saline or Ang II infusion mice (n = 6 per group).** NS, no statistical significance. *t* test.
(TIF)

Figure S2 **Negative antibody was replaced by control IgG.** (**A**) Representative immunohistochemical staining of IL-6 expression in WT mice with antibody aganist IL-6 or control IgG. (**B**) Representative Immunohistochemical staining of α-SMA expression in WT mice with antibody aganist α-SMA or control IgG. Scale bars: 50 µm.
(TIF)

Figure S3 **Immunofluorescence staining of IL-6 (green) and F4/80 (red) expressions in Ang II-treated hearts of WT mice.** Scale bars: 50 µm.
(TIF)

Figure S4 **Negative controls of Cultured macrophages and myofibroblasts induce α-SMA, TGF-β1 and Smad3 expression.** (**A**) Representative immunohistochemical staining of α-SMA with 3-D system with WT or IL-6-/- macrophages and WT or IL-6-/- fibroblasts in the present of Ang II. (**B**) Immunofluorescence staining of protein level of phosphorylated Smad3 (p-Smad3; green) and DDR2 (red) or Immunofluorescence staining of TGF-β1 (green) and DDR2 (red) in cultured WT fibroblasts and IL-6-/- fibroblasts. Scale bars: 50 µm. (n = 6 per group). Scale bars: 50 µm.
(TIF)
