# Supplementary figures and images for: Macrophage-Stimulated Cardiac Fibroblast Production of IL-6 Is Essential for TGF β/Smad Activation and Cardiac Fibrosis Induced by Angiotensin II
Source: PLoS One. 2012 May 4;7(5):e35144. doi: 10.1371/journal.pone.0035144 (PMC3344835; doi:10.1371/journal.pone.0035144)

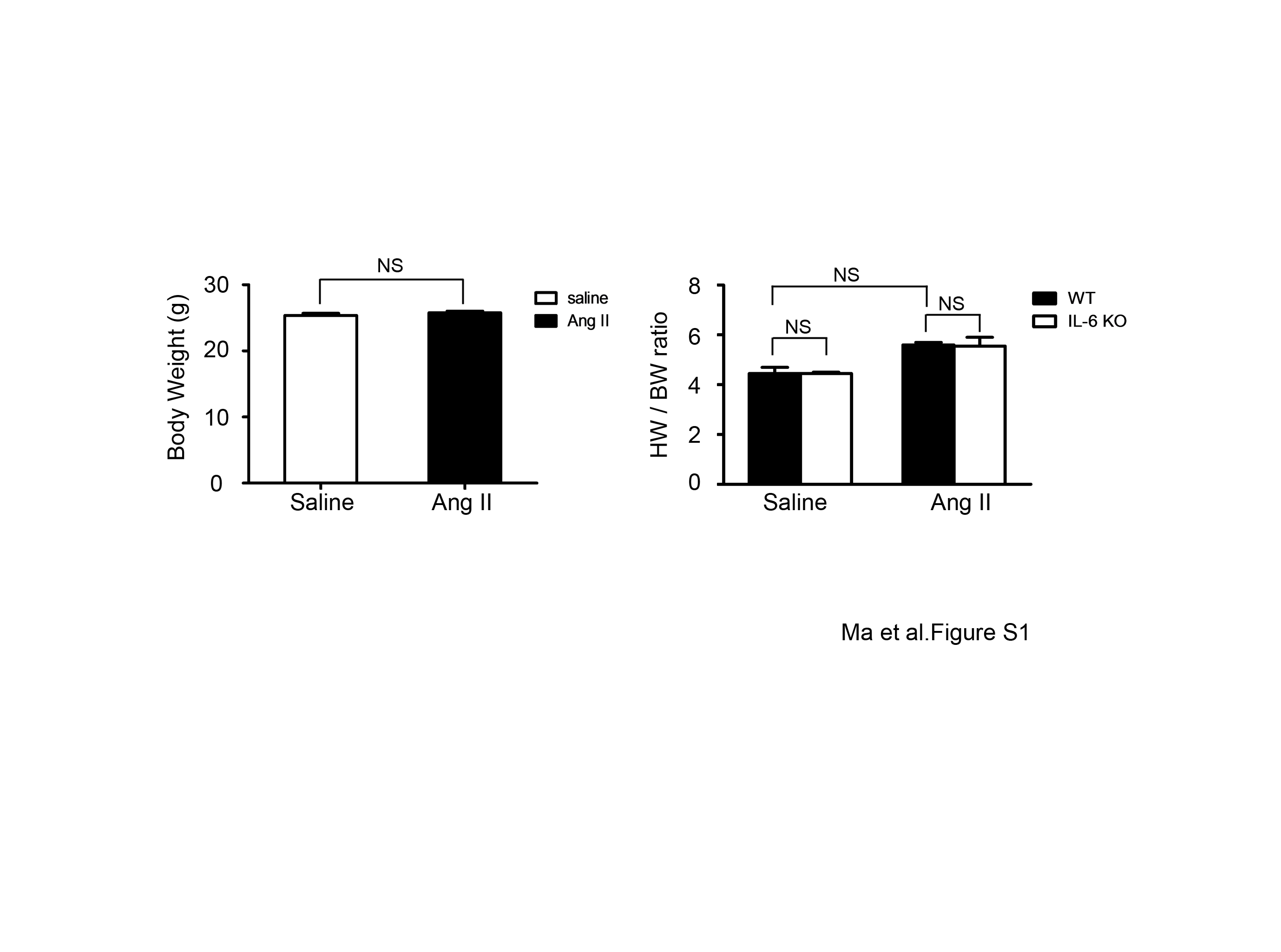

Supplement: Figure S1 — Quantification of body weight and heart/body weight from saline or Ang II infusion mice (n = 6 per group). NS, no statistical significance. t test. (TIF) [file pone.0035144.s001.tif]

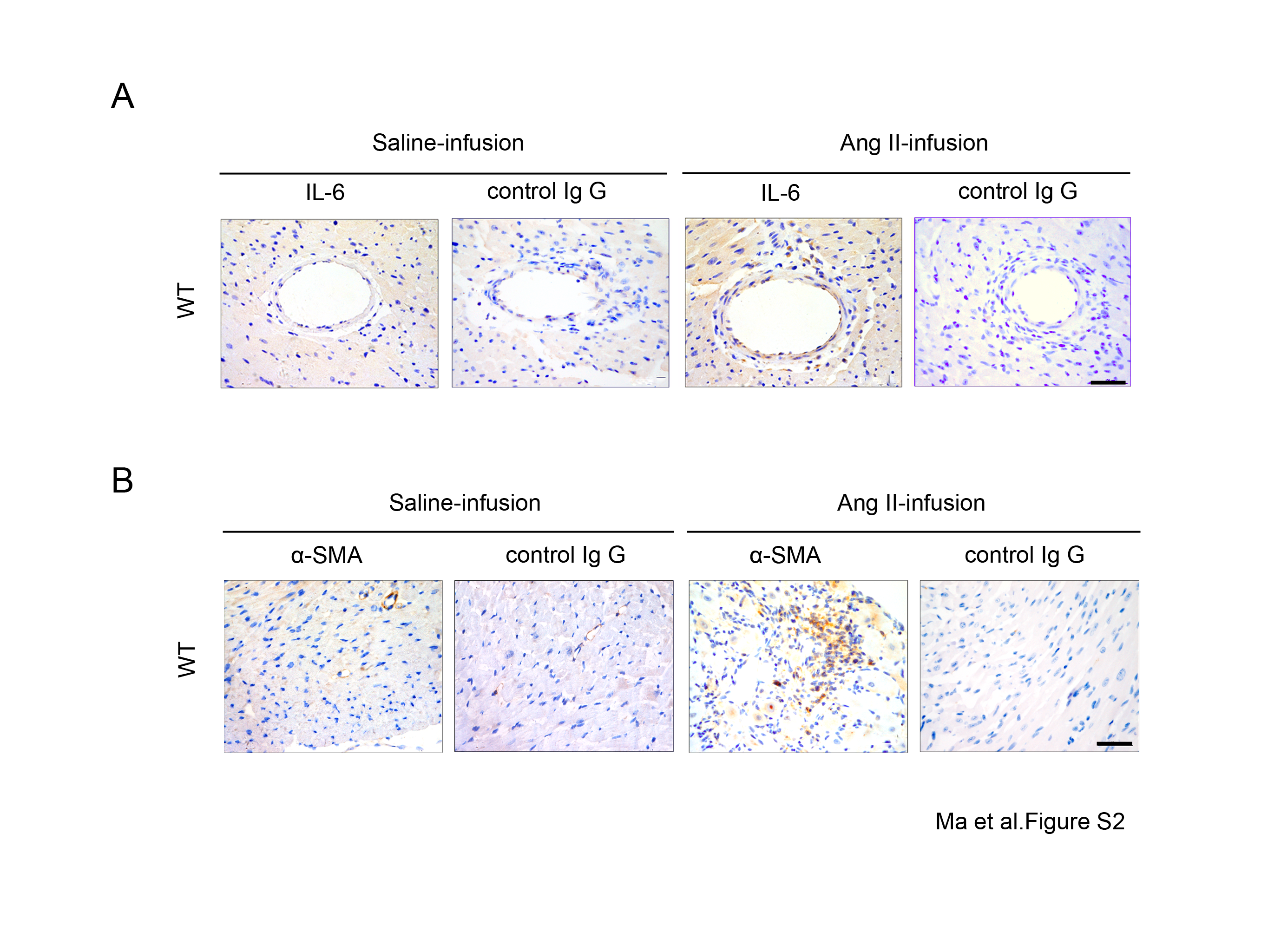

Supplement: Figure S2 — Negative antibody was replaced by control IgG. (A) Representative immunohistochemical staining of IL-6 expression in WT mice with antibody aganist IL-6 or control IgG. (B) Representative Immunohistochemical staining of α-SMA expression in WT mice with antibody aganist α-SMA or control IgG. Scale bars: 50 µm. (TIF) [file pone.0035144.s002.tif]

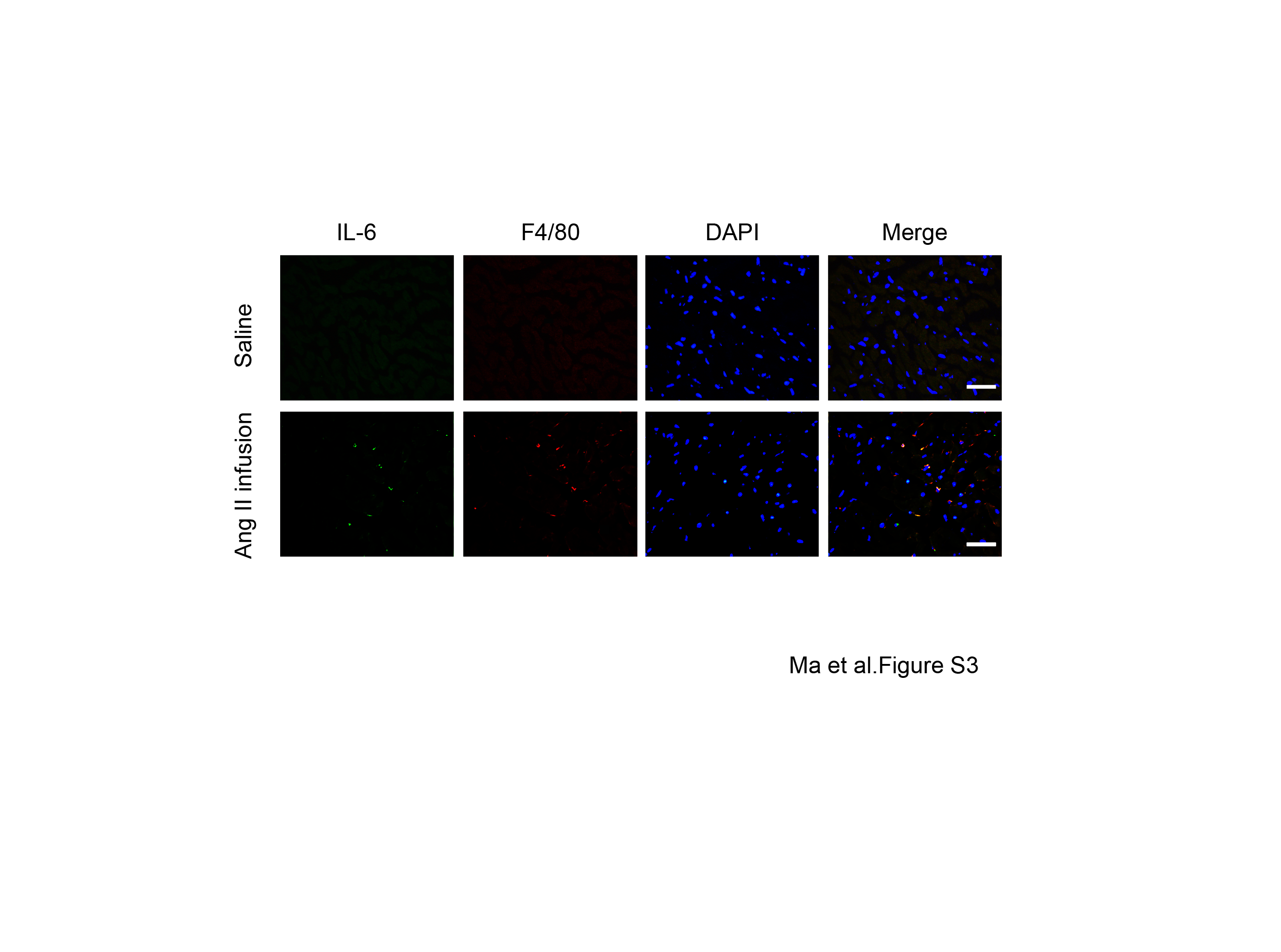

Supplement: Figure S3 — Immunofluorescence staining of IL-6 (green) and F4/80 (red) expressions in Ang II-treated hearts of WT mice. Scale bars: 50 µm. (TIF) [file pone.0035144.s003.tif]

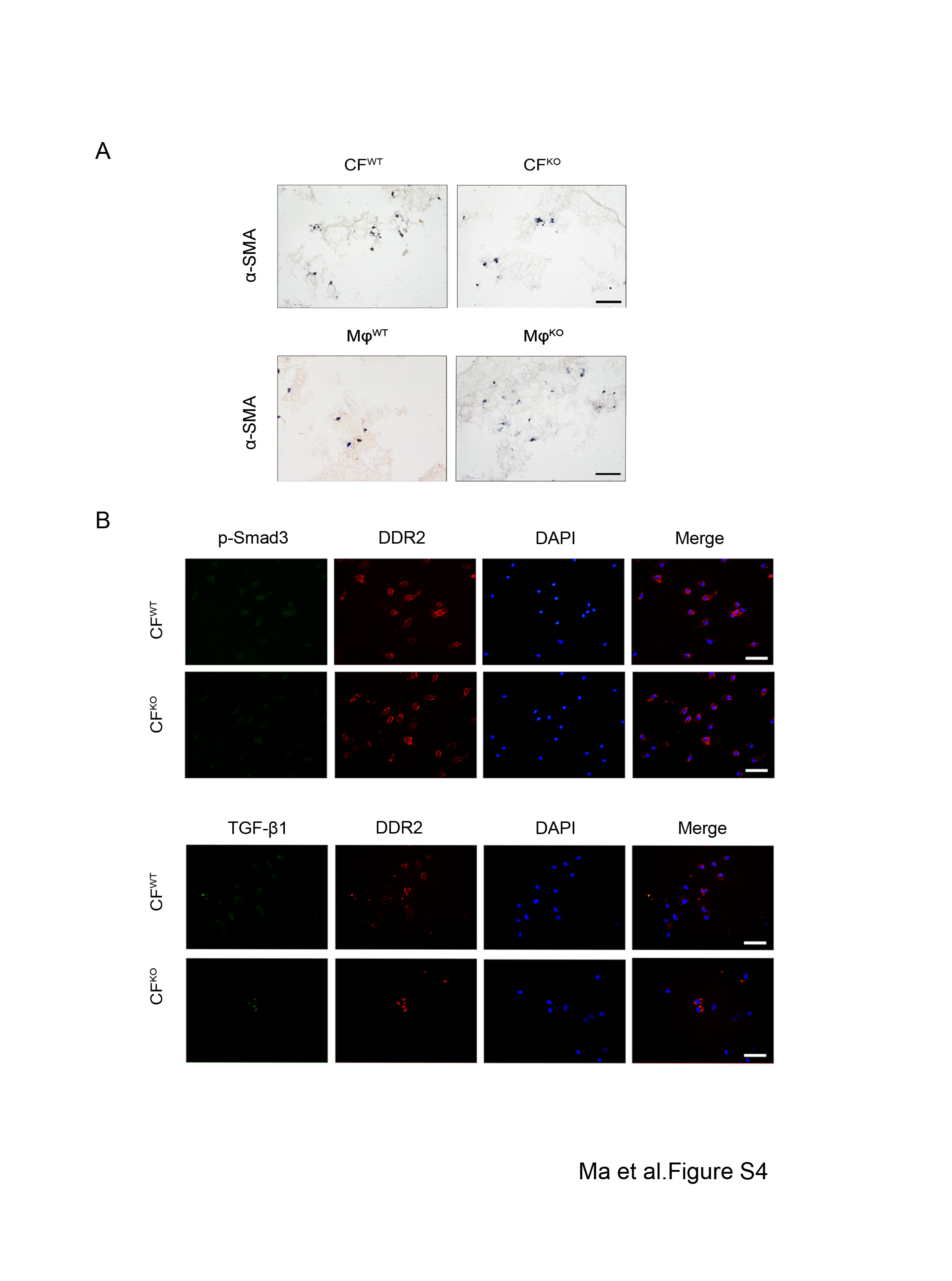

Supplement: Figure S4 — Negative controls of Cultured macrophages and myofibroblasts induce α-SMA, TGF-β1 and Smad3 expression. (A) Representative immunohistochemical staining of α-SMA with 3-D system with WT or IL-6-/- macrophages and WT or IL-6-/- fibroblasts in the present of Ang II. (B) Immunofluorescence staining of protein level of phosphorylated Smad3 (p-Smad3; green) and DDR2 (red) or Immunofluorescence staining of TGF-β1 (green) and DDR2 (red) in cultured WT fibroblasts and IL-6-/- fibroblasts. Scale bars: 50 µm. (n = 6 per group). Scale bars: 50 µm. (TIF) [file pone.0035144.s004.tif]
